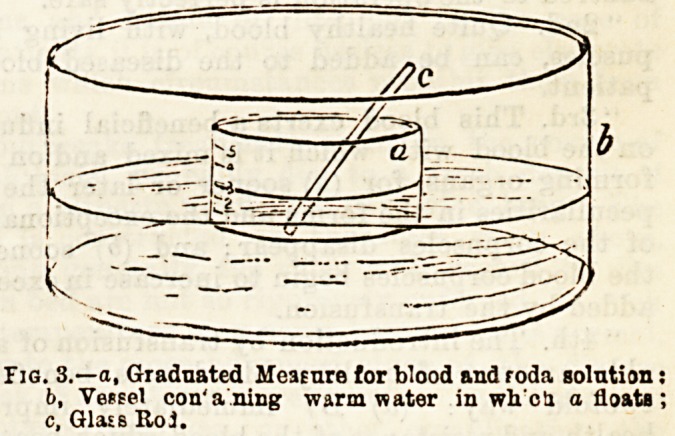# On the Treatment of Pernicious Anæmia by Transfusion of Blood

**Published:** 1893-07-15

**Authors:** 


### THE HOSPITAL CLINIC.

[*The Editor will be glad to receive offers of co-operation and contributions from members of the profession. All letters should be addressed to* THE EDITOR, THE LODGE, PORCHESTER SQUARE, LONDON, W.]

### ROYAL INFIRMARY, EDINBURGH.

### ON THE TREATMENT OF PERNICIOUS ANÆMIA BY TRANSFUSION OF BLOOD.

Any light thrown on the treatment of the comparatively hopeless class of cases to which pernicious anæmia belongs cannot but be welcome. Dr. D. T. Brakenridge, in conjunction with his colleague, Mr. John Duncan, of this hospital, has recently obtained excellent results by the frequent transfusion of blood from healthy subjects into the veins of the sufferer. In all of their cases the ordinary medicinal treatment with iron, arsenic, &c., had been employed, with its usual variable and uncertain results, and the transfusion was resorted to when the life of the patient seemed in great danger. In the absence of a definite knowledge of the pathology of the condition—whether it is due to some fault in the blood-forming tissues, or to over activity of the blood-destroying organs, or to a combination of these, or, on the other hand, to a definite *ptomain* or poison—treatment cannot be otherwise than empirical to a greater or less extent. The results of this method of treatment appear to Dr. Brakenridge to throw a side light on the pathology, and he believes "that pernicious anæmia is mainly due to a faulty genesis of the corpuscles in the blood-forming organs, and a consequent tendency to their early death in the blood-destroying organs."

FIG. 1.—*a*, Nozzle, with *b*, Short Rubber Tube.

FIG. 2.—Syringe.

He therefore argued that repeated transfusions of healthy blood would at least "give such an impetus to the blood-forming organs as would bring them within the influence of the curative action of arsenic."

*Technique of Operation.*—The operation, although a comparatively simple one, requires great attention to details. The chief points to ensure success are (1) perfect asepsis, (2) normal temperature of the blood, (3) fluidity of the blood, (4) slow injection of the blood. In order to avoid all risk of septic infection, the apparatus used must be as simple as possible Mr. Duncan uses a small pen shaped glass nozzle (Fig. 1, *a*), about the size of a No. 6 catheter, for introducing into the vein of the receiver. To the end of this about two inches of india-rubber tubing (*b*) is firmly tied with silk. A glass syringe (Fig. 2), with a nozzle fitting the tubing, and holding about four ounces of fluid, completes the injecting apparatus. The blood flowing from the donor is caught in a small flat graduated glass vessel (Fig. 3, *a*), which floats in warm water contained in a larger vessel of the same shape (*b*). A glass rod (*c*) is also required. All these are thoroughly cleansed in strong carbolic lotion, and then washed with water which has been rendered aseptic by boiling. Other ordinary antiseptic precautions are scrupulously observed.

A vein in the arm of the receiver is exposed, a double ligature passed under it, the lower thread tied, the vein opened, and the pen-shaped nozzle introduced and fixed. The blood is drawn from the donor, and to prevent it coagulating, is received in the graduated vessel which contains a definite quantity of a 5 per cent. solution of sodium phosphate, with which it is mixed by stirring with the glass rod. The proportion is one part of soda-solution to three parts of blood. The mixture is kept at the temperature of the body by floating the vessel in warm water, and the syringe is surrounded by boracic lint wrung out of warm water.

The strength of the soda-solution is of importance. If it be too strong it destroys the red blood corpuscles

FIG. 3.—*a*, Graduated Measure for blood and soda solution; *b*, Vessel containing warm water in which *a* floats; *c*, Glass Rod.

if too weak the blood coagulates and is lost. It is made with boiled distilled water.

Mr. Duncan lays great stress on the importance of introducing the mixture very slowly into the circulation. For a considerable time the heart and blood vessels have adapted themselves to a greatly diminished quantity of fluid, and suddenly and rapidly to introduce six or eight ounces under these conditions would undoubtedly interfere with the heart's action. The introduction of eight ounces of fluid should occupy about thirty minutes. Pain in the back, and marked increase in the force of the heart's action, indicate that the injection is being performed too rapidly.

The operation is repeated at short intervals till the red corpuscles and hæmoglobin approximate to their normal proportions.

It is needless to say that the blood must be taken from persons in perfect health. In Dr. Brakenridge's cases the donors were students attached to his wards, and they were all in particularly good condition, in most cases the red corpuscles being above the normal.

*Results*—In all the cases observed the transfusion was followed very rapidly by a marked increase in the number of red blood corpusles, and this increase progressed, with slight variations, after every subsequent operation till a healthy standard was reached. In one case the patient was *in extremis* when the operation was performed, and died soon afterwards, but not before the red blood corpuscles had increased 100,000 per c.m.m. The following brief summary of five of Dr. Brakenridge's cases will indicate the results of this method of treatment:—

| | Red Blood Corpuscles per c.m.m. | Hæmoglobin per cent. | Remarks. |
|---|---|---|---|
| Case I. | | | |
| Before transfusion... | 1,160,000 | 24 | |
| 7 hours after ,, ... | 1,470,000 | | |
| 4½ months later ...... (5 transfusions) | 3,000,000 | 60 | |
| Case II. | | | |
| Before transfusion... | 640,000 | 20 | Patient quite well 3½ years after. |
| 7 days after ,, ... | 2,080,000 | | |
| 4 months later ...... | 4,000,000 | 75 | |
| Case III. | | | |
| Before transfusion... | 480,000 | 26 | Died next day. |
| 24 hours after ,, ... | 580,000 | | |
| Case IV. | | | |
| Before transfusion... | 920,000 | | Still under treatment when case reported. |
| 7 hours after ,, ... | 1,110,000 | | |
| 5 months later ...... | 1,770,000 | | |
| Case V. | | | |
| Before transfusion... | 890,000 | 11 | |
| After ,, ... | 930,000 | 13 | |
| 52 days later ......... | 4.200,000 | 54 | |

In another patient operated on by Mr. Duncan for Dr. J. O. Affleck, between February 24th and April 21st, the red corpuscles increased from 1,410,000 to 5,110,000.

Dr. Brakenridge sums up his opinion of the procedure as follows:—

"1st. If all the necessary precautions are strictly adhered to the operation is perfectly safe.

"2nd. Quite healthy blood, with living blood corpuscles, can be added to the diseased blood of the patient.

"3rd. This blood exerts a beneficial influence, both on the blood with which it is mixed and on the blood-forming organs, for (*a*) sooner or later the abnormal peculiarities in the forms and the exceptional varieties of the corpuscles disappear; and (*b*) sooner or later the blood corpuscles begin to increase in excess of those added by the transfusion.

"4th. The introduction by transfusion of a considerable amount of healthy blood acts beneficially in a twofold way: (*a*) By immediately improving the health and resistance of the blood which becomes mixed with it; and (*b*) later on, by gradually operating beneficially on the blood-forming organs through which it circulates, restoring their blood-forming functions to the normal condition."

## Figures and Tables

**Fig. 1. f1:**
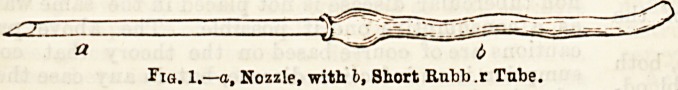


**Fig. 2. f2:**
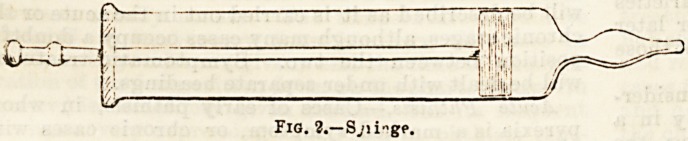


**Fig. 3. f3:**